# Autochthonous transmission of extensively drug-resistant *Neisseria gonorrhoeae* in Germany, 2025

**DOI:** 10.2807/1560-7917.ES.2025.30.40.2500738

**Published:** 2025-10-09

**Authors:** Regina Selb, Hana Tlapák, Kathleen Klaper, Susanne Buder, Klaus Jansen, Dagmar Heuer

**Affiliations:** 1 Robert Koch Institute, Unit of HIV/AIDS, STI and Blood-borne Infections, Department of Infectious Disease Epidemiology, Berlin, Germany; 2 Robert Koch Institute, Unit of Sexually Transmitted Bacterial Pathogens and HIV, Department of Infectious Diseases, Berlin, Germany; 3 Robert Koch Institute, German Reference Laboratory for Gonococci, Unit of Sexually Transmitted Bacterial Pathogens, Department of Infectious Diseases, Berlin, Germany; * These authors contributed equally to this work and share first authorship.; ** These authors contributed equally to this work and share last authorship.

**Keywords:** XDR, *Neisseria gonorrhoeae*, resistance, autochthonous transmission, Germany, penA 60.001, sexually transmitted infections

## Abstract

We report four confirmed autochthonously transmitted gonorrhoea cases in Germany in 2025, with *Neisseria gonorrhoeae* isolates exhibiting high-level azithromycin and cefixime resistance. Regarding ceftriaxone, isolates were susceptible for two cases and resistant for two, rendering the later extensively drug-resistant (XDR-NG). Cases, all heterosexual males, were in their late teens to mid-60s. Three isolates were MLST ST18091, with the mosaic *penA*-60.001 allele and the 23S rRNA A2045G mutation and were related to an XDR-NG detected in the United Kingdom in 2024.

Extensively drug-resistant (XDR) *Neisseria gonorrhoeae* (NG) strains are of great public health concern and emerging in Europe [1-3]. We describe XDR-NG acquisitions by confirmed autochthonous transmission in Germany. These events were detected following notification of two gonorrhoea cases in the country in 2025. Another two cases, which are presented in this report as well, also resulted from autochthonous transmission, but were confirmed multidrug-resistant (MDR) NG. Among the four notified cases, a strong molecular connection was confirmed between three with respective available isolates. All infections occurred in heterosexual men with symptomatic urethritis who lived in the same geographical region.

## Extensively drug-resistant gonorrhoea in Germany up to 2025

In Germany, gonorrhoea cases with reduced susceptibility to azithromycin, cefixime or ceftriaxone are notifiable to the Robert Koch Institute (RKI) according to §7.3 of the German Infection Protection Act (IfSG) [4]. Additionally, (NG)-isolates are submitted to the German national routine Gonococci Resistance Surveillance programme (Go-Surv-AMR) on a voluntary basis. In 2023 and 2024, one gonorrhoea case with XDR-NG per year was diagnosed and notified in the country [5]. Intensified surveillance indicated that these cases remained single and unconnected, with travel history to South-East Asia confirmed or suspected.

## Clinical and epidemiological case descriptions

In 2025, a similar case to those in the 2 years prior was reported (i.e. confirmed travel history to South East Asia), but four additional cases, all in the federal state of North Rhine-Westphalia, did not report recent travel and are further described.

### Case 1

The first of the four cases was notified to the RKI in the beginning of April 2025 due to resistance to ceftriaxone and cefixime and high-level resistance to azithromycin. The corresponding NG-isolate was sent to the RKI in the framework of Go-Surv-AMR. In March 2025, the case, a male in his late teens, had consulted the emergency department of a hospital because of a symptomatic urethritis. He stated that contact to a female sex worker in Germany was the most probable route of transmission. The case was treated with a combination of ceftriaxone (2 g intravenously (i.v.); single dose) and azithromycin (1.5 g per os (p.o.); single dose). Test of cure 4 weeks after treatment was successful.

### Case 2

The second case was also notified in the beginning of April 2025. Because of the finding of resistance to ceftriaxone, cefixime and azithromycin, the corresponding isolate was sent to the RKI for confirmation of the resistant phenotype. In March 2025, the man aged in his mid-40s had consulted a private urology practice with a symptomatic urethritis. This practice was in geographical proximity to the hospital that case 1 had attended. He declared having had sexual contact with a female sex worker in Germany. He was treated with ceftriaxone for 3 days (2 g i.v.; daily). Test of cure 4 weeks after the end of treatment was successful.

### Case 3

The third case was notified in March 2025 due to the finding of cefixime resistance and high level-resistance to azithromycin. The isolate was reported as susceptible to ceftriaxone and sent to the RKI for confirmation of its resistance profile, but could not be revived. The man in his mid-60s had consulted a private urology practice in geographical proximity to the healthcare facilities visited by cases 1 and 2, also due to symptomatic urethritis. He stated to have had heterosexual contact in Germany and reported no recent travel history; contact of case 3 with a sex worker was suspected by the treating physician. The case was treated with a combination of ceftriaxone (2 g intramuscular (i.m.); single dose) and azithromycin (1.5 g p.o.; single dose) and subsequently declared to be free of symptoms. However, the case preferred not to undergo test of cure.

### Case 4

The fourth case was notified in the beginning of July 2025 with NG-resistance to cefixime and ceftriaxone and high-level resistance to azithromycin and the isolate was sent to the RKI. The male in his late teens visited the emergency department of the same hospital as case 1 during a night shift because of symptomatic urethritis. He reported to have had sexual contact with a woman who was not his steady female partner. The patient received a prescription for ceftriaxone to retrieve at the pharmacy and was supposed to return to the hospital for i.v. application of the antibiotic in the morning. However, he did not show up for the appointment and did not come back to the hospital even after repeated calls by the medical staff.

## Microbiological investigation of viable isolates

At the RKI, all isolates, except the one of case 3, were successfully cultured on PolyViteX agar (BioMérieux, France). Minimum inhibitory concentrations (MICs) for six antimicrobials were determined by Etest (BioMérieux, France) and interpreted according to breakpoints recommended by the European Committee on Antimicrobial Susceptibility Testing (EUCAST) version 15.0 [6]. Presence of β-lactamase was determined with nitrocefin test (Becton Dickinson, Heidelberg, Germany).

All three isolates showed high-level resistance to azithromycin and were resistant to cefixime, ciprofloxacin, and tetracycline (Table). They were susceptible to increased exposure of penicillin and did not produce a β-lactamase. Isolates of cases 1 (G25–563) and 2 (G25-K71) were also resistant to ceftriaxone and were therefore classified as XDR. The XDR/MDR-definitions of the European Gonococcal Antimicrobial Surveillance Programme (Euro-GASP) are currently being revised. We defined isolates as XDR when resistance to cefixime and ceftriaxone, a MIC value > 1 mg/L regarding azithromycin, and resistance to at least two further substances (ciprofloxacin, penicillin, spectinomycin or tetracycline) are confirmed (personal communication: Magnus Unemo, October 2025). The reported ceftriaxone-resistance of the isolate of case 4 (G25–1113) could not be confirmed at the RKI, where the strain was found susceptible, so it was classified as MDR-NG. The case 3 isolate that could not be revived had been reported to the RKI as displaying high level-azithromycin resistance as well as cefixime resistance (MIC: 1 mg/L), while being susceptible to ceftriaxone (MIC: 0.125 mg/L). This corresponded to an isolate bearing an MDR-NG strain.

**Table:** *Neisseria gonorrhoeae* XDR/MDR isolates’ MICs according to central retesting at the RKI and data on treatment of cases, Germany, March−July2025 (n = 4 cases)

Antimicrobial drug	G25-563 (case 1)		G25-K71 (case 2)		Case 3	G25-1113 (case 4)	
	MIC in mg/L	Interpretation (EUCAST v 15.0)	MIC in mg/L	Interpretation (EUCAST v 15.0)		MIC in mg/L	Interpretation (EUCAST v 15.0)
Azithromycin	> 256	Resistant (high-level)	> 256	Resistant (high-level)	Not viable^a^	> 256	Resistant (high-level)
Cefixime	1	Resistant	1	Resistant		1	Resistant
Ceftriaxone	0.19	Resistant	0.19	Resistant		0.125	Susceptible
Ciprofloxacin	4	Resistant	3	Resistant		4	Resistant
Penicillin	0.25	Susceptible to increased exposure	0.25	Susceptible to increased exposure		0.25	Susceptible to increased exposure
Tetracycline	32	Resistant (high-level)	32	Resistant (high-level)		24	Resistant (high-level)
Collection date	March 2025		March 2025		March 2025	June 2025	
Treatment	2 g ceftriaxone (i.v.) single dose; 1.5 g azithromycin (p.o.) single dose		2 g ceftriaxone (i.v.) daily for 3 days		2 g ceftriaxone (i.m.) single dose; 1.5 g azithromycin (p.o.) single dose	Unclear	
Test of cure (4 weeks after end of treatment)	Cured		Cured		Not performed, free of symptoms	Unclear	

EUCAST: European Committee on Antimicrobial Susceptibility Testing; i.m.: intramuscular; i.v.: intravenous; MIC: minimum inhibitory concentration; p.o.: per os; RKI: Robert Koch Institute; XDR: extensively drug-resistant.

^a^ An isolate for this case was sent to the RKI but could not be successfully cultured.

## Molecular investigation

From the isolates’ cultures, DNA was extracted with a QIAcube Connect (Qiagen) and the DNeasy Blood and Tissue QIAcube Kit (Qiagen), and whole genome sequencing (WGS) was performed as previously described [7]. Raw sequencing data were submitted to the European Nt Archive (ENA, Accession number: PRJEB97528). Read qualities and de novo assembly were assessed using an in-house pipeline using GARI v1.1.1 (https://github.com/rki-mf1/GARI). Multilocus sequence typing (MLST), NG multi antigen sequence typing (NG-MAST), NG sequence typing for antimicrobial resistance (NG-STAR) and resistance gene prediction were performed using the Pathogenwatch platform. Previously identified, travel-associated XDR-NG genetic sequences from Germany and the sequencing data of nine European XDR strains [1-3], including the WHOQ strain [8], were also uploaded to the platform for generation of a core-single-nt polymorphism distance-based neighbour-joining phylogenetic tree [9].

Isolates G25-563, G25-K71 and G25-1113 were assigned to MLST ST18091, NG-MAST ST22862 and NG-STAR ST5793. All three isolates harboured the mosaic *penA*-60.001 allele causing reduced susceptibility to the extended spectrum cephalosporins cefixime and ceftriaxone [10]. The high-level resistance to azithromycin was conferred by the A2045G mutation of all four alleles of the 23S rRNA-encoding gene [11]. Presence of the *tetM* gene and the mutation in the *rpsJ* gene conferring the V57M substitution accounted for the high-level resistance to tetracycline [11].

Isolates G25-563 and G25-K71 were separated by only one single nt polymorphism (SNP), while isolate G25-1113 differed from each of these two isolates by three and two SNPs, respectively. The closest related European XDR-NG was found to be the strain H24-496 from the United Kingdom, reported in 2024, which was linked to travel to Cambodia (Figure) [3]. On the phylogenetic tree these four isolates formed a separate cluster from the other European XDR-NG and the previously identified XDR-NG from Germany, which were not closely related to the isolates linked with autochthonous transmission in Germany.

**Figure:**
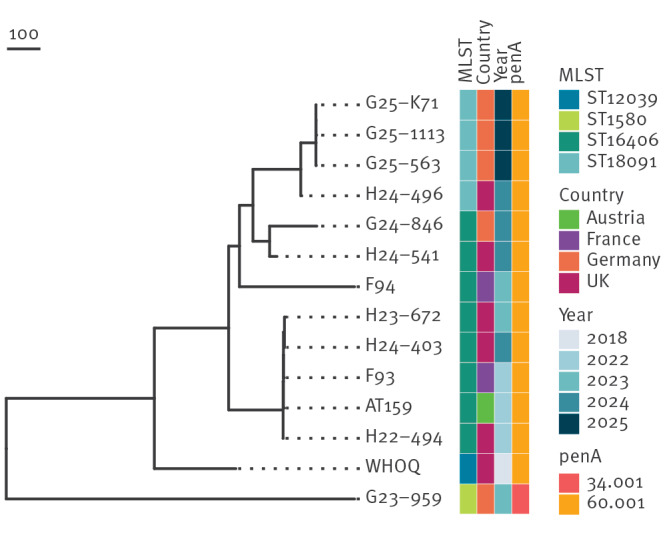
Phylogenetic relationships among published sequences of European XDR *Neisseria gonorrhoeae* isolates, including the sequences of newly characterised isolates G25–563, G25-K71, and G25-1113 from Germany, April−July 2025

MLST: multi locus sequence typing; UK: United Kingdom.

## Discussion

We describe occurrences of the first known gonorrhoea cases with autochthonous transmission of XDR/MDR-NG in Germany. Currently these events seem restricted to heterosexual men with contact to sex work in a confined geographical area. Despite one case occurring 4 months after the initial case of a series of four, the close molecular relationship among three of the four respective isolates that could be analysed, suggests a prolonged transmission of the specific pathogen, potentially with contact to the same currently unknown index case in the context of sex work. 

The strains autochthonously transmitted in Germany belonged to MLST ST18091, a single allele variant of ST16406 which is the most prevalent ST for XDR-NG isolated in Europe [3]. The presence of these strains in Germany shows further geographical spread of XDR-isolates combining ceftriaxone resistance conferred by the mosaic *penA*-60.001 allele with high-level azithromycin resistance caused by the 23S rRNA A2045G mutation. 

Nevertheless, three patients were successfully treated with a dose of 2 g ceftriaxone as monotherapy or in combination with 1.5 g azithromycin in accordance with current treatment guidelines in Germany. This was successful because of relatively low ceftriaxone MICs despite resistance, suggesting that ceftriaxone in higher doses (1–2 g) may be an effective treatment option in this setting [12,13]. The success of treatment may also depend on the site of infection, as most known treatment failures involved pharyngeal infections [14-17]. In cases of resistance, individual therapy consultation by specialised sexually transmitted infection (STI) practitioners and reference laboratories, taking into account the results of antibiotic susceptibility tests and tests of cure, is crucial for further therapy management.

Our study has some limitations. Additional autochthonous transmission events may have occurred in the country, which were not notified because susceptibility testing was not performed. Indeed, currently, only gonorrhoea cases with confirmed reduced susceptibility to azithromycin, cefixime and ceftriaxone are notifiable to the RKI and culture is also a prerequisite for sending isolates to Go-Surv-AMR. Furthermore, asymptomatic infections which are more prevalent among women and in extragenital sites such as the pharynx and anal region may remain undiagnosed and thus contribute to ongoing transmission. 

As part of continuous efforts to prevent NG spread, we informed a broad range of stakeholders regarding prevention, diagnostic and treatment of NG both on the regional and national level and emphasized the importance of easily accessible STI-testing and treatment options including susceptibility testing and test of cure. In North Rhine-Westphalia, local health authorities offer anonymous STI-testing and treatment for sex workers and other key populations free of charge. In addition, at national level the reference laboratory at the RKI provides diagnostic and therapeutic advice to physicians in cases of NG resistance. 

## Conclusion

The emergence and autochthonous spread of XDR-NG in Germany threaten the effectiveness of ceftriaxone for treating gonorrhoea. Public health measures must focus on offering easily accessible STI-testing and treatment options in order to stop infection chains among sex workers and their clients and also to prevent spread into other key populations vulnerable to STI like men who have sex with men (MSM) or young people. Together with the mandatory notification of NG, Go-Surv-AMR as part of an integrated genomic surveillance (IGS) approach remains indispensable in Germany for detailed characterisation of NG with unusual resistance, to identify closely related transmission events and to provide an overview of the national resistance situation. 

## Data Availability

The genomic data for this study has been deposited in the European Nucleotide Archive (ENA) at EMBL-EBI under accession number PRJEB97528 (https://www.ebi.ac.uk/ena/browser/view/PRJEB97528).
